# Interpretation of medium resolution cryoEM maps of multi-protein complexes

**DOI:** 10.1016/j.sbi.2019.06.009

**Published:** 2019-10

**Authors:** Ana Casañal, Shabih Shakeel, Lori A Passmore

**Affiliations:** MRC Laboratory of Molecular Biology, Cambridge CB2 0QH, United Kingdom

## Abstract

Electron cryo-microscopy (cryoEM) is used to determine structures of biological molecules, including multi-protein complexes. Maps at better than 3.0 Å resolution are relatively straightforward to interpret since atomic models of proteins and nucleic acids can be built directly. Still, these resolutions are often difficult to achieve, and map quality frequently varies within a structure. This results in data that are challenging to interpret, especially when crystal structures or suitable homology models are not available. Recent advances in mass spectrometry techniques, computational methods and model building tools facilitate subunit/domain fitting into maps, elucidation of protein contacts, and *de novo* generation of atomic models. Here, we review techniques for map interpretation and provide examples from recent studies of multi-protein complexes.

## Highlights

• CryoEM maps at medium (3.5–6 Å) resolution can be challenging to interpret.
• Integration of multiple methods can inform cryoEM studies.
• Mass spectrometry and biochemistry facilitate map interpretation and model building.

**Current Opinion in Structural Biology** 2019, **58**:166–174

This review comes from a themed issue on **CryoEM**

Edited by **Matteo D Peraro** and **Ji-Joon Songo**

For a complete overview see the Issue and the Editorial

Available online 27th July 2019

**https://doi.org/10.1016/j.sbi.2019.06.009**

0959-440X/© 2019 MRC Laboratory of Molecular Biology. Published by Elsevier Ltd. This is an open access article under the CC BY license (http://creativecommons.org/licenses/by/4.0/).

## Introduction

Atomic models are essential for understanding the molecular mechanisms of biological processes. Advances in electron cryo-microscopy (cryoEM) have enabled the elucidation of 3D reconstructions and atomic models of specimens whose structure determination was not feasible only a few years ago. Still, challenges limit the resolution that can be achieved in many cases. For example, difficulties in making suitable specimens, compositional and conformational heterogeneity, and complex stability limit the quality of cryoEM maps. This results in challenges in generating reliable 3D reconstructions, identifying subunits in large assemblies, and building atomic models.

By combining cryoEM with other structural biology techniques and biochemical, biophysical, and mass spectrometry-based methods, it is possible to gain more insight into the mechanism of many complexes. Models generated using this integrative structural biology method can be used to test functional predictions (both *in vivo* and *in vitro*) and thereby address specific mechanistic questions.

There are many excellent recent reviews on cryoEM specimen preparation and 3D reconstruction [1, 2, 3, 4, 5] and we will not cover these here. Here, we review recent integrative approaches used to build models from medium resolution (3.5–6 Å) cryoEM maps of macromolecular complexes.

## Map quality

Advances in cryoEM software have allowed improved reconstructions of structurally dynamic complexes. One of the major advances has been the implementation of a statistical framework [6] during refinement. This statistical framework has now been extended to pre-processing steps such as per particle motion correction (Bayesian polishing in *RELION* [7]) and per particle CTF refinement [8^••^]. Additionally, some of the microscope misalignments (beam tilt) as well as Ewald sphere can be corrected [7,9, 10, 11].

Approaches to refine flexible regions within dynamic complexes have also been implemented, including signal subtraction followed by focused classification or focused refinement for different areas of the map [10,12,13]. This multi-step process has recently been combined into a single task by multi-body refinement [14^•^]. Principal component analysis can then identify the underlying motions present in the complex. The accessibility and ease-of-use of cryoEM software have also greatly improved [8^••^,^10^,^15^].

**Figure 1:**
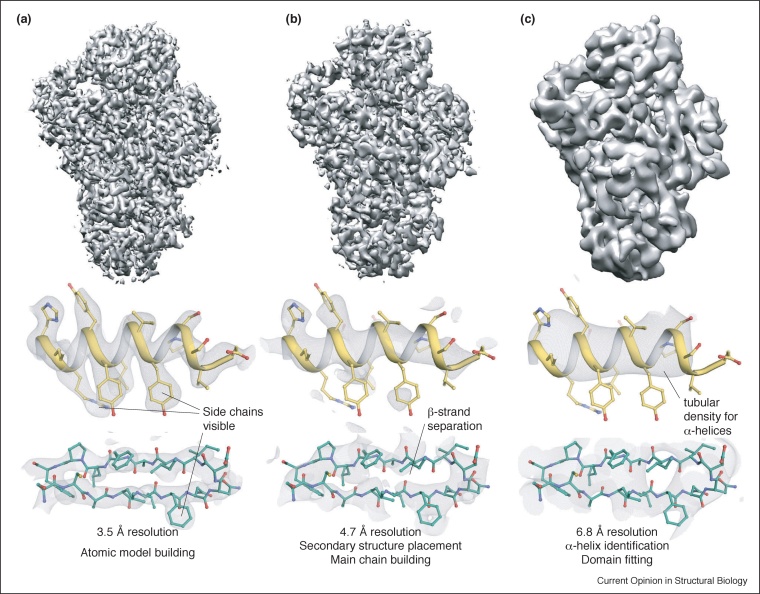
Visualization of structural features at different resolutions. The polymerase module of the Cleavage and Polyadenylation Factor (CPF) [19^••^] was reconstructed from different numbers of particles to give B-factor sharpened maps at 3.5 Å **(a)**, 4.7 Å **(b)**, or 6.8 Å **(c)** resolution. The overall reconstructions are shown in surface representation (top). Alpha helical (middle) and beta strand (bottom) regions of the maps with models are also shown.

The resolution and quality of a cryoEM map determine the level of biological interpretation that is feasible. Structures with resolutions better than 2.5 Å have good side chain density and atomic models can be built directly into the maps, but these have been determined for only a small number of proteins [16, 17, 18]. With expertise, *de novo* model-building can be performed at resolutions up to ∼3.8 Å because the backbone and large side chains are visible. At lower resolutions, different structural features are apparent: beta strands are separated at resolutions better than ∼4.5 Å, and alpha helices are resolved as tubular densities at resolutions better than 8 Å (Figure 1).

Visualization of higher resolution features allows a more detailed interpretation of maps (Figure 1). Still, even at relatively low resolutions (6–10 Å), known crystal structures can be positioned within a map with high accuracy, and alpha helical models can be built, giving important functional insight. Notably, the overall resolution of a structure does not imply that all regions can be interpreted equally. Local resolution maps [20] are useful for estimating resolution variability, but manual visual inspection is essential for assessing map quality.

**Figure 2:**
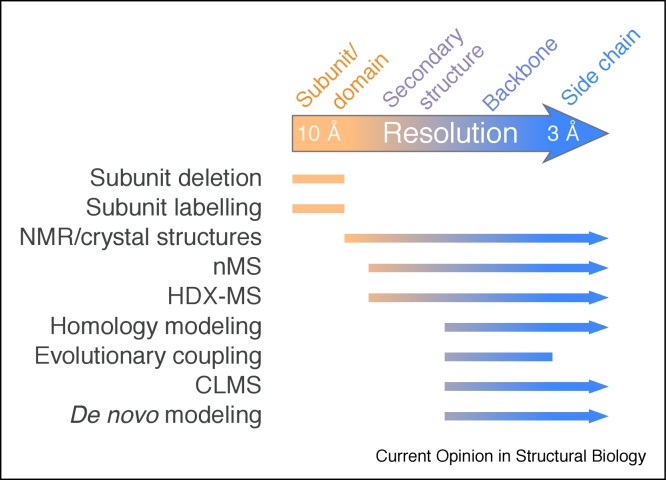
Multi-resolution modeling of structures of multi-protein complexes. A selection of methods used in integrative structural biology along with features that can be modeled at different resolutions are shown. Arrows represent the resolution range where highlighted methods are useful. nMS, native mass spectrometry; HDX-MS, hydrogen-deuterium exchange mass spectrometry; CLMS, cross-linking mass spectrometry.

Despite improvements in sample preparation, data collection and computational methods, often the resolution of a cryoEM structure does not go beyond 3.5 Å. Fortunately, even if the specimen cannot be improved biochemically [21] and the map quality cannot be improved with additional data collection and processing, other methods can be used to help interpret maps (Figure 2). Below, we describe such strategies.

## Methods to assist with subunit identification

The first step in interpreting medium resolution (3.5–6 Å) cryoEM maps of multi-protein complexes is to identify the locations of individual subunits. In some cases, high-resolution structures are available, for example, from X-ray crystallography, and these can be fit into the maps, initially using rigid body fitting. In recent studies of the yeast Cleavage and Polyadenylation Factor (CPF), the characteristic shape of a beta propeller subunit was visible, allowing us to locate the position of the Pfs2 subunit [19^••^]. In another example, a crystal structure of the dimerization domain of the cytoplasmic motor protein dynein was fit into a cryoEM structure of the dynein tail bound to its cofactor dynactin, to explain how dynein's two chains are held together [22^•^].

Subunits can also be located within cryoEM maps by labelling strategies. For example, imaging of complexes after adding a bulky tag, binding of an antibody, or deletion of a specific subunit can be used to identify its location. In a recent 10 Å resolution structure of the COMPASS complex, a globular eGFP tag [23], the rod-like dynein light chain-interacting domain (DID) [24], and a high affinity Fab-epitope tag (PA-NZ) [25] were used as subunit-specific labelling strategies to determine the overall subunit organization [26^•^]. In a 4.0 Å resolution map of the dynactin complex [27^•^], previous rotary-shadowed EM images of antibody-labelled subunits [28] helped to identify subunit locations. In another example, analysis of the 500 kDa core CPF complex in the presence and absence of the Ysh1 nuclease subunit allowed identification of the position of Ysh1 within a low-resolution negative stain map [29^••^].

## Mass spectrometry

**Figure 3:**
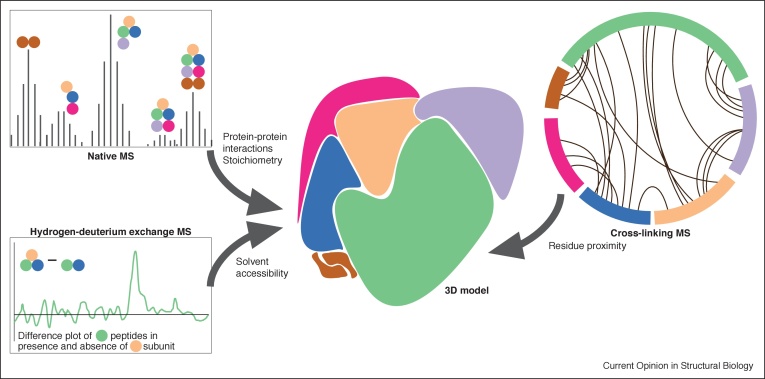
Interpretation of cryoEM maps of multi-protein complexes using mass spectrometry. Mass spectrometry (MS) can be instrumental in interpreting medium-resolution cryoEM maps. Types of MS and the information they yield are shown.

Mass spectrometry (MS) has emerged as one of the most powerful techniques to complement structural biology. It can reveal information about the stoichiometry and composition of protein complexes, interaction surfaces, dynamic regions and the presence of small molecules [30]. Importantly, it can provide information on all parts of a complex, including those that are less well-ordered or not visible in cryoEM structures. There are several types of MS that are used in combination with cryoEM, including native MS, hydrogen-deuterium exchange MS, and cross-linking MS (Figure 3) [31].

### Native mass spectrometry

Native MS (nMS) is used to analyze intact protein complexes [32,33^•^]. Because the protein assemblies are preserved in the gas phase, intact masses can be determined, and therefore subunit stoichiometries can be calculated. For example, nMS was used to determine subunit stoichiometry in a 17 Å resolution structure of RNA polymerase II bound to a capping enzyme [34]. An elegant study of the Kai circadian oscillator used nMS to guide cryoEM sample preparation, to assist with model building, and to verify the structural models using mutational analysis [35^••^]. nMS can also be used to identify ligands, such as lipids [33^•^].

Protein complexes can be dissociated into subcomplexes in the mass spectrometer. After analysis of the resulting subcomplexes/subunits by nMS and computational network analysis, it is possible to build a protein–protein interaction network of multi-component assemblies. We recently used nMS to elucidate a subunit interaction map of CPF [19^••^]. The complex had been initially purified as a fifteen-subunit complex, but nMS revealed that only fourteen subunits were part of CPF. The fifteenth protein was part of a separate complex with overlapping subunits called APT. Further analysis of the nMS data revealed that CPF comprises three modules, each incorporating one of the enzymatic activities. These nMS data were used to define appropriate purification protocols for CPF subcomplexes, and for subunit identification in cryoEM maps.

### Hydrogen-deuterium exchange mass spectrometry

Hydrogen-deuterium exchange MS (HDX-MS) can define the dynamics and interaction interfaces of proteins within a complex [36]. HDX-MS involves exposure of a protein to deuterium oxide (D_2_O), resulting in rapid exchange of hydrogen for deuterium. After the exchange reactions are quenched at different time points, proteins are digested and the relative quantities of hydrogen and deuterium can be measured by MS. This is most informative for backbone amide hydrogens and occurs faster in disordered or exposed regions (those not involved in stable hydrogen bonding interactions) than in structured regions. The rate of exchange can provide information on secondary structure, protein–protein interactions, ligand binding sites and conformational changes. HDX-MS is particularly useful in comparative studies. For example, interaction surfaces and conformational changes can be mapped by HDX-MS analysis of proteins in the presence and absence of a binding partner. This reveals peptides that are protected or exposed upon interaction.

HDX-MS has been successfully used to assist in the interpretation of cryoEM maps. For example, it was used to confirm interfaces between subunits of the Kai circadian oscillator derived from a cryoEM map at 4.7 Å resolution [35^••^]. Also, it has been used to examine the conformation, dynamics and ligand binding of insulin degrading enzyme [37] and the Hsp104 AAA+ ATPase [38].

### Cross-linking mass spectrometry

Chemical crosslinking coupled to MS (CLMS) can be used to identify protein segments that are in close spatial proximity within macromolecular complexes [39,40^•^,41^•^]. In CLMS, pairs of functional groups (most commonly the primary amine group in lysines) are covalently cross-linked. The reaction products are then enzymatically digested, cross-linked peptides are enriched, analyzed by MS, and identified by database searching. CLMS therefore reveals residues in close proximity within and between protein subunits. These can be used as distance restraints when building atomic models into cryoEM maps and to define conformational changes [39,40^•^,^42^].

CLMS has been used in many studies to confirm, guide or actively model protein structures in cryoEM maps. For instance, CLMS was crucial in generating models of the nuclear pore complex in combination with other structural data including low resolution maps from cryo-electron tomography [43, 44, 45,46^••^]. It was also important for generating a structure of the 26S proteasome [47], an RNA polymerase II – Mediator complex [48^•^] and the mammalian mitochondrial complex I [49]. For CPF, CLMS defined contacts between proteins that were not visible by cryoEM and crystallography [19^••^].

## Homology modeling, structure prediction and evolutionary covariance

To generate initial models, homology modelling of domains, subunits or heteromeric complexes can be very helpful. Since structure is usually more conserved than sequence, homology modelling can be useful even for remote homologs. Recent methods combine sequence alignments, predicted secondary structure and template-based modeling to generate new models. If distance constraints are available, for example from CLMS, these can sometimes be used as restraints. A number of programs are available to generate homology models, including *PHYRE2* [50], *I-TASSER* [51], *SWISS-MODEL* [52], *Robetta* [53], and *MODELLER* [54]. Webservers make these programs easily accessible.

Other computational tools can also be extremely useful. For example, evolutionary covariance of individual residues detects correlated evolutionary sequence changes to identify amino acids that are likely to form direct contacts [55,56]. This requires the availability of a sufficient number of diverse sequences. In studies of bovine mitochondrial ATP synthase, evolutionary covariance analysis allowed model building for one of the subunits into cryoEM maps at subnanometer resolution [57^•^]. Programs/webservers include *EVcouplings/EVcomplex* and *Gremlin* [55,56].

## Subunit modeling

All structural and biochemical data can be combined to generate (atomic) models that agree with the cryoEM structure of a multi-protein complex. In some cases, existing structures or models can be fit into the map but if that is not possible, *de novo* modelling can be performed [58^••^]. This can be challenging and is best performed by integrating data from multiple sources.

### Initial models

At resolutions worse than 3 Å, automatic *de novo* model-building programs typically result in incomplete solutions. Still, methods are improving and programs such as *ARP/wARP* [59], *buccaneer* [60], *Rosetta* [61^••^], *phenix.map_to_model* [62], *pathwalking* [63] and *EMBuilder* [64] are in development. Some cryoEM projects that have successfully integrated initial models generated by these programs include amyloid fibrils (*ARP/wARP EM*, [65]), the imidazoleglycerol-phosphate dehydratase (IGPD) enzyme of the histone biosynthesis pathway (*buccaneer* and *Rosetta* [66]), Paramecium bursaria chlorella virus 1 (PBCV-1), viral RNA-polymerase, and mechanosensitive ion channels (*EMbuilder*, [67, 68, 69]).

Molecular replacement methods, such as *MOLREP* [58^••^,^70^], or *RosettaES* [71] can be used for density-based fold recognition using a database of known protein domains/fragments. These can provide suitable templates for *de novo* model building or automated rebuilding [72].

### Model building and refinement

*Coot* [58^••^,^73^] has been extensively used for both crystallography and cryoEM model-building and it now provides a set of improved tools for model building into cryoEM maps including morphing, jiggle fit, Cα baton-mode, *de-novo* model building (including helices, beta-strands, RNA and DNA), loop fitting, and local distance restraints. For example, one can combine jiggle fitting and morphing of a domain with placing idealized secondary structure elements (SSEs, alpha helices and beta strands) and baton-building of new main chain atoms.

Helices and beta-strands can be first identified in a given map either visually or with the aid of the ‘Find Secondary Structure’ functionality followed by their automatic building using the ‘Place Helix Here’ or ‘Place Strand Here’ tools. The SSE and modeled domains can then be refined using appropriate alpha helix, beta-strand, ProSMART [74^•^] or Rama restraints [75]. By using secondary structure prediction, it may be possible to determine how the SSEs are connected to each other when the resolution in those regions does not allow complete visualization of connecting loops.

Next, if the map offers enough resolution to identify the side chains of some residues, these can be used as starting points for amino acid sequence assignment. It is important to ensure that the amino acid chemistry makes sense, for example, residues fit within appropriate hydrophobic and hydrophilic environments. Constraints from other methods (e.g. CLMS) should also be satisfied. After tracing the density and building the structure of an unknown domain, one can use the model to search the PDB or the DALI server [76] for homologous structures that may further facilitate model building.

When manually inspecting maps, it is often useful to simultaneously view multiple blurred/sharpened maps which can be generated using a combination of *Coot* and *REFMAC* [74^•^]. This facilitates interpretation of intermediate resolution maps as features of the main chain (blurring) and side-chains (sharpening) can both be visualized with more detail. Confidence maps [77] and locally sharpened maps [78] may also assist model-building.

Once a round of model building has come to an end, models are typically refined with *REFMAC5* in Fourier space [79] or *phenix.real_space_refine* in real space [80]. At intermediate resolutions, *Rosetta* [72,81] provides an automated approach for *de novo* model building and to improve the geometry and sidechain placement of atomic models. Other fitting and refinement programs available are *Cryo-Fit* [82], *MDFF* [83,84], *DireX* [85] and *iMODFIT* [86].

### Validation and assessment

Validation of atomic models is essential. Validation tools are available in *Coot*, *Molprobity* and *EMRinger* [87, 88, 89] to analyze geometry, density fit, rotamers, and Ramachandran outliers. These can identify problematic regions that can be modified or improved to ensure the fitness of the final model. It is often the case that not all outliers can be fixed. If a sequence can be reliably assigned but density for side chains is not apparent, side chains can be removed (stubbed) from the models. If the sequence cannot be assigned, models can be built as poly-alanine. Model quality can also be assessed using a recently described multi-model approach [90].

### Integrative modelling

Although manual or semi-automated methods may work for interpretation of many cryoEM datasets at medium resolution, methods also exist to computationally integrate many different types of data simultaneously. These can provide a more unbiased and comprehensive approach. For example, models can be generated using distance restraints from CLMS, subunit stoichiometries and connectivities from nMS, and subunit locations from labelling, mutational and deletion experiments. Computational approaches to integrate such data include integrative modelling platform (IMP, [91]), HADDOCK [92,93] and XL-MOD [94].

## Conclusions and future perspectives

**Figure 4:**
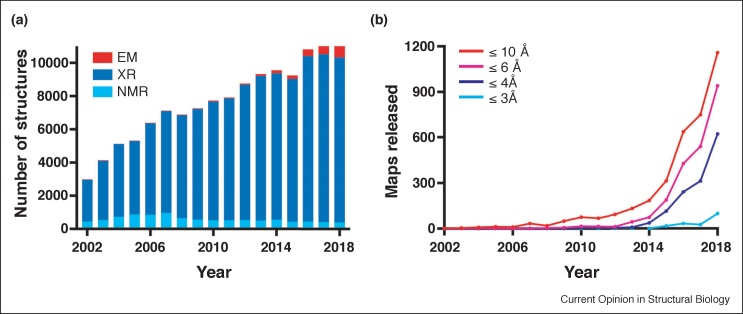
Number of structures and maps released in the PDB and EMDB. **(a)** Number of structures released in the PDB from NMR, X-ray crystallography (XR) and cryoEM between 2002 and 2018. The number of cryoEM structures has rapidly increased since 2013. **(b)** EMDB map releases at given resolutions between 2002 and 2018. Of a total of 7885 maps deposited in the EMDB at the submission time of this manuscript, 1587 (20%) and 246 (3%) have a resolution better than 4 Å and 3 Å, respectively. In 2018, 1770 maps were released in the EMDB, 624 (35%) and 98 (5.5%) achieved a resolution better than 4 Å and 3 Å, respectively.

Recent progress in cryoEM has led to a substantial increase in the number of cryoEM structures that can be interpreted with atomic models (Figure 4). Still, many structures are of medium resolution and are therefore more difficult to interpret (Figure 4b). Integrative methods allow multi-resolution modelling, including regions that cannot be visualized by cryoEM because they are flexibly tethered within complexes. Additional new methods, for example, using manifold embedding to map continuous conformational changes [95], will facilitate our understanding of the molecular motions within dynamic complexes.

The interplay between cryoEM and crystallography may become increasingly important, with cryoEM guiding construct design for crystallization and crystal structures becoming invaluable to interpreting cryoEM maps (especially for small domains or regions where cryoEM does not lead to high enough resolution). The building of atomic models into maps of 3.5–6 Å resolution remains a challenge but additional developments in MS, computational methods, automatic model generation, and *de novo* model building will continue to improve cryoEM structure determination. More efforts towards integrating information from diverse experimental and theoretical data will simplify and speed-up interpretation of the structures of macromolecular complexes. Importantly, integrative methods not only facilitate interpretation of cryoEM maps by providing a more complete description of protein complex architecture and assembly, but they also broaden our biochemical and mechanistic understanding of cellular machines.

## Conflict of interest statement

Nothing declared.

## References and recommended reading

Papers of particular interest, published within the period of review, have been highlighted as:

• of special interest
•• of outstanding interest
